# Crystal structure of *catena*-poly[[(3-*tert*-butyl­pyridine-κ*N*)(4-*tert*-butyl­pyridine-κ*N*)cadmium]-di-μ-thio­cyanato-κ^2^ *N*:*S*;κ^2^ *S*:*N*]

**DOI:** 10.1107/S1600536814024647

**Published:** 2014-11-19

**Authors:** Julia Werner, Thorben Reinert, Inke Jess, Christian Näther

**Affiliations:** a Institut für Anorganische Chemie, Christian-Albrechts-Universität Kiel, Max-Eyth-Strasse 2, 24118 Kiel, Germany; b Institut für Physikalische Chemie, Christian-Albrechts-Universität Kiel, Max-Eyth-Strasse 1, 24118 Kiel, Germany

**Keywords:** crystal structure, cadmium coordination polymer, μ-1,3-thio­cyanate anions

## Abstract

In the crystal structure of the title compound, [Cd(NCS)_2_(C_9_H_13_N)_2_]_*n*_, the Cd^II^ cations are coordinated in a slightly distorted octa­hedral geometry by one 3-*tert*-butyl­pyridine ligand, one 4-*tert*-butyl­pyridine ligand and two pairs of translationally-equivalent μ-1,3-bridging thio­cyanate ligands, all of which are in general positions. These μ-1,3-bridging thio­cyante anions bridge the Cd^II^ cations, forming chains that propagate parallel to the *b* axis.

## Related literature   

For related crystal structures with μ-1,3-bridging thio­cyanate anions, see: Banerjee *et al.* (2005 ▶); Reinert *et al.* (2012*a* ▶,*b* ▶); Tahli *et al.* (2011 ▶).
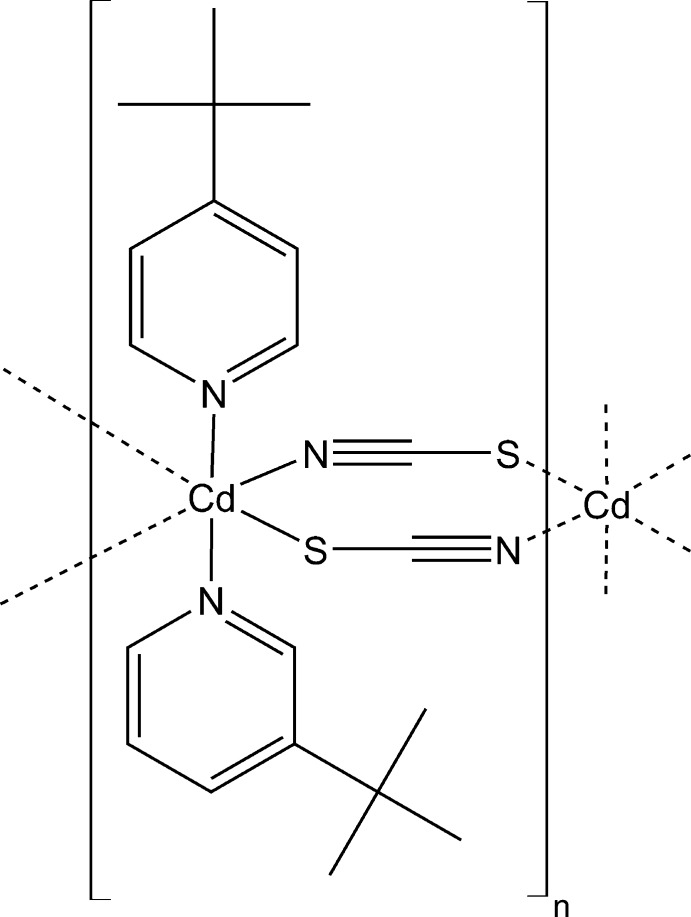


## Experimental   

### Crystal data  

[Cd(NCS)_2_(C_9_H_13_N)_2_]
*M* *_r_* = 498.97
Monoclinic, 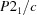

*a* = 20.0917 (8) Å
*b* = 5.9503 (2) Å
*c* = 21.0198 (9) Å
β = 115.902 (3)°
*V* = 2260.51 (15) Å^3^
*Z* = 4
Mo *K*α radiation
μ = 1.16 mm^−1^
*T* = 293 K
0.16 × 0.12 × 0.09 mm

### Data collection  

STOE IPDS-2 diffractometer
Absorption correction: numerical (*X-SHAPE* and *X-RED32*; Stoe & Cie, 2008 ▶) *T*_min_ = 0.760, *T*_max_ = 0.895
25365 measured reflections
5407 independent reflections
4159 reflections with *I* > 2σ(*I*)
*R*_int_ = 0.044

### Refinement  

*R*[*F* ^2^ > 2σ(*F* ^2^)] = 0.046
*wR*(*F* ^2^) = 0.084
*S* = 1.14
5407 reflections
244 parameters
H-atom parameters constrained
Δρ_max_ = 0.41 e Å^−3^
Δρ_min_ = −0.50 e Å^−3^

### 

Data collection: *X-AREA* (Stoe & Cie, 2008 ▶); cell refinement: *X-AREA*; data reduction: *X-AREA*; program(s) used to solve structure: *SHELXS97* (Sheldrick, 2008 ▶); program(s) used to refine structure: *SHELXL97* (Sheldrick, 2008 ▶); molecular graphics: *XP* in *SHELXTL* (Sheldrick, 2008 ▶) and *DIAMOND* (Brandenburg, 2011 ▶); software used to prepare material for publication: *publCIF* (Westrip, 2010 ▶).

## Supplementary Material

Crystal structure: contains datablock(s) I, global. DOI: 10.1107/S1600536814024647/pk2535sup1.cif

Structure factors: contains datablock(s) I. DOI: 10.1107/S1600536814024647/pk2535Isup2.hkl

**Fig. 1.**
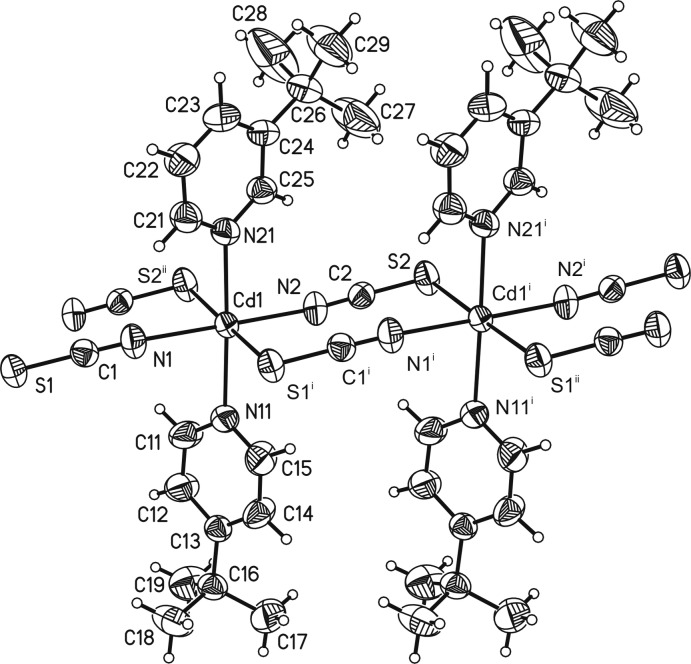
: Crystal structure of the title compound. Displacement ellipsoids are drawn at the 50% probability level. Symmetry code: i: (x, y+1, z); ii: (x, y-1, z).

. DOI: 10.1107/S1600536814024647/pk2535fig1.tif
Crystal structure of the title compound. Displacement ellipsoids are drawn at the 50% probability level. Symmetry code: i: (x, y+1, z); ii: (x, y-1, z).

CCDC reference: 1033510

Additional supporting information:  crystallographic information; 3D view; checkCIF report

## supplementary crystallographic information

### S1. Synthesis and crystallization

CdSO_4_·8/3H_2_O was purchased from Merck, and 4-*tert*-butyl­pyridine and Ba(NCS)_2_·3H_2_O were purchased from Alfa Aesar. The 4-*tert*-butyl­pyridine has a purity of only 97% and is contaminated with 3-*tert*-butyl­pyridine, which cannot be separated. Cd(NCS)_2_ was synthesized by stirring 17.5 g (57.00 mmol) Ba(NCS)_2_·3H_2_O and 14.6 g (57.00 mmol) CdSO_4_·8/3H_2_O in 300 mL H_2_O at RT for three hours. The white residue of BaSO_4_ was filtered off and the solvent was evaporated by heating. The homogeneity of the product was investigated by X-ray powder diffraction and elemental analysis. The title compound was prepared by the reaction of (0.15 mmol) 34.3 mg Cd(NCS)_2_ and (0.15 mmol) 22.2 µL 4-tert-butyl­pyridine in 1.0 mL H_2_O at 80 °C in a closed 10 mL glass culture tube. After several days, colorless crystalline blocks were obtained.

### S2. Refinement

Carbon-bound H atoms were positioned with idealized geometry and refined with *U*_iso_(H) = 1.2 *U*_eq_(C) using a riding model with C—H = 0.95 Å for aromatic and C—H = 0.99 Å for methyl H atoms.

### Crystal data

**Table:**

[Cd(NCS)_2_(C_9_H_13_N)_2_]	*F*(000) = 1016
*M**_r_* = 498.97	*D*_x_ = 1.466 Mg m^−^^3^
Monoclinic, *P*2_1_/*c*	Mo *K*α radiation, λ = 0.71073 Å
Hall symbol: -P 2ybc	Cell parameters from 25365 reflections
*a* = 20.0917 (8) Å	θ = 2.0–28.0°
*b* = 5.9503 (2) Å	µ = 1.16 mm^−^^1^
*c* = 21.0198 (9) Å	*T* = 293 K
β = 115.902 (3)°	Block, colourless
*V* = 2260.51 (15) Å^3^	0.16 × 0.12 × 0.09 mm
*Z* = 4	

### Data collection

**Table:**

STOE IPDS-2 diffractometer	5407 independent reflections
Radiation source: fine-focus sealed tube	4159 reflections with *I* > 2σ(*I*)
Graphite monochromator	*R*_int_ = 0.044
ω scans	θ_max_ = 28.0°, θ_min_ = 2.0°
Absorption correction: numerical (*X-SHAPE* and *X-RED32*; Stoe & Cie, 2008)	*h* = −26→26
*T*_min_ = 0.760, *T*_max_ = 0.895	*k* = −7→7
25365 measured reflections	*l* = −27→27

### Refinement

**Table:**

Refinement on *F*^2^	Primary atom site location: structure-invariant direct methods
Least-squares matrix: full	Secondary atom site location: difference Fourier map
*R*[*F*^2^ > 2σ(*F*^2^)] = 0.046	Hydrogen site location: inferred from neighbouring sites
*wR*(*F*^2^) = 0.084	H-atom parameters constrained
*S* = 1.14	*w* = 1/[σ^2^(*F*_o_^2^) + (0.0326*P*)^2^ + 0.8678*P*] where *P* = (*F*_o_^2^ + 2*F*_c_^2^)/3
5407 reflections	(Δ/σ)_max_ = 0.001
244 parameters	Δρ_max_ = 0.41 e Å^−^^3^
0 restraints	Δρ_min_ = −0.50 e Å^−^^3^

### Special details

**Table:**

Geometry. All esds (except the esd in the dihedral angle between two l.s. planes) are estimated using the full covariance matrix. The cell esds are taken into account individually in the estimation of esds in distances, angles and torsion angles; correlations between esds in cell parameters are only used when they are defined by crystal symmetry. An approximate (isotropic) treatment of cell esds is used for estimating esds involving l.s. planes.
Refinement. Refinement of F^2^ against ALL reflections. The weighted R-factor wR and goodness of fit S are based on F^2^, conventional R-factors R are based on F, with F set to zero for negative F^2^. The threshold expression of F^2^ > 2sigma(F^2^) is used only for calculating R-factors(gt) etc. and is not relevant to the choice of reflections for refinement. R-factors based on F^2^ are statistically about twice as large as those based on F, and R- factors based on ALL data will be even larger.

### Fractional atomic coordinates and isotropic or equivalent isotropic displacement parameters (Å^2^)

**Table:**

	*x*	*y*	*z*	*U*_iso_*/*U*_eq_	
Cd1	0.732991 (14)	0.24172 (4)	0.269228 (13)	0.04067 (8)	
N1	0.67220 (19)	0.5638 (5)	0.20986 (17)	0.0535 (7)	
C1	0.65872 (17)	0.7440 (6)	0.18809 (16)	0.0415 (6)	
S1	0.63714 (5)	0.99805 (14)	0.15449 (5)	0.0484 (2)	
N2	0.79392 (17)	−0.0756 (5)	0.32949 (17)	0.0514 (7)	
C2	0.80555 (17)	−0.2557 (6)	0.35212 (16)	0.0421 (6)	
S2	0.82427 (6)	−0.51157 (15)	0.38460 (5)	0.0554 (2)	
N11	0.81232 (17)	0.2863 (5)	0.21360 (15)	0.0482 (7)	
C11	0.8505 (3)	0.4716 (7)	0.2206 (2)	0.0724 (13)	
H11	0.8473	0.5835	0.2501	0.087*	
C12	0.8948 (3)	0.5105 (7)	0.1870 (2)	0.0713 (13)	
H12	0.9209	0.6449	0.1950	0.086*	
C13	0.9014 (2)	0.3547 (6)	0.14183 (19)	0.0443 (7)	
C14	0.8606 (2)	0.1617 (7)	0.1342 (3)	0.0638 (11)	
H14	0.8622	0.0474	0.1047	0.077*	
C15	0.8174 (2)	0.1363 (7)	0.1698 (2)	0.0638 (11)	
H15	0.7901	0.0047	0.1623	0.077*	
C16	0.9479 (2)	0.3988 (7)	0.1019 (2)	0.0509 (8)	
C17	0.9747 (3)	0.1778 (9)	0.0830 (3)	0.0847 (15)	
H17A	1.0038	0.2105	0.0580	0.127*	
H17B	0.9328	0.0882	0.0536	0.127*	
H17C	1.0043	0.0967	0.1255	0.127*	
C18	0.8990 (3)	0.5207 (10)	0.0334 (3)	0.0867 (16)	
H18A	0.9269	0.5508	0.0072	0.130*	
H18B	0.8821	0.6598	0.0444	0.130*	
H18C	0.8571	0.4282	0.0056	0.130*	
C19	1.0150 (3)	0.5424 (10)	0.1453 (3)	0.0862 (16)	
H19A	1.0432	0.5674	0.1191	0.129*	
H19B	1.0452	0.4664	0.1888	0.129*	
H19C	0.9990	0.6840	0.1557	0.129*	
N21	0.64660 (16)	0.2045 (5)	0.31532 (16)	0.0470 (7)	
C21	0.5826 (2)	0.3142 (7)	0.2852 (2)	0.0546 (9)	
H21	0.5733	0.4039	0.2459	0.066*	
C22	0.5296 (2)	0.2999 (7)	0.3098 (2)	0.0629 (11)	
H22	0.4855	0.3795	0.2879	0.076*	
C23	0.5429 (2)	0.1660 (7)	0.3673 (2)	0.0573 (10)	
H23	0.5075	0.1554	0.3846	0.069*	
C24	0.60838 (19)	0.0467 (6)	0.39999 (18)	0.0454 (8)	
C25	0.6582 (2)	0.0755 (6)	0.37141 (19)	0.0473 (8)	
H25	0.7030	−0.0005	0.3928	0.057*	
C26	0.6255 (2)	−0.0980 (7)	0.4648 (2)	0.0587 (10)	
C27	0.6837 (4)	−0.2738 (12)	0.4745 (4)	0.133 (3)	
H27A	0.6662	−0.3706	0.4339	0.200*	
H27B	0.6933	−0.3611	0.5160	0.200*	
H27C	0.7285	−0.2011	0.4797	0.200*	
C28	0.6512 (6)	0.0518 (13)	0.5280 (3)	0.171 (4)	
H28A	0.6627	−0.0373	0.5696	0.257*	
H28B	0.6127	0.1569	0.5223	0.257*	
H28C	0.6945	0.1319	0.5327	0.257*	
C29	0.5577 (3)	−0.2282 (11)	0.4579 (4)	0.1055 (19)	
H29A	0.5416	−0.3255	0.4175	0.158*	
H29B	0.5188	−0.1249	0.4521	0.158*	
H29C	0.5699	−0.3165	0.4997	0.158*	

### Atomic displacement parameters (Å^2^)

**Table:**

	*U*^11^	*U*^22^	*U*^33^	*U*^12^	*U*^13^	*U*^23^
Cd1	0.04773 (12)	0.03305 (11)	0.04745 (12)	0.00141 (12)	0.02654 (10)	0.00495 (11)
N1	0.064 (2)	0.0352 (15)	0.0566 (18)	0.0003 (14)	0.0216 (16)	0.0037 (14)
C1	0.0434 (15)	0.0405 (15)	0.0407 (15)	−0.0062 (17)	0.0185 (13)	−0.0032 (17)
S1	0.0550 (5)	0.0374 (4)	0.0470 (5)	0.0007 (4)	0.0167 (4)	0.0063 (4)
N2	0.0536 (18)	0.0355 (14)	0.0603 (19)	0.0036 (13)	0.0204 (15)	0.0053 (14)
C2	0.0422 (15)	0.0421 (16)	0.0425 (15)	−0.0014 (17)	0.0190 (13)	−0.0044 (17)
S2	0.0675 (6)	0.0385 (4)	0.0481 (5)	−0.0007 (4)	0.0141 (5)	0.0058 (4)
N11	0.0579 (17)	0.0447 (16)	0.0456 (15)	−0.0034 (14)	0.0260 (14)	−0.0023 (13)
C11	0.108 (4)	0.055 (2)	0.084 (3)	−0.028 (2)	0.070 (3)	−0.024 (2)
C12	0.097 (3)	0.057 (2)	0.086 (3)	−0.032 (2)	0.065 (3)	−0.021 (2)
C13	0.0418 (18)	0.0475 (18)	0.0463 (19)	0.0013 (15)	0.0218 (16)	0.0042 (15)
C14	0.069 (3)	0.055 (2)	0.090 (3)	−0.017 (2)	0.055 (3)	−0.023 (2)
C15	0.063 (2)	0.052 (2)	0.088 (3)	−0.017 (2)	0.044 (2)	−0.011 (2)
C16	0.048 (2)	0.059 (2)	0.053 (2)	0.0025 (17)	0.0279 (17)	0.0068 (17)
C17	0.087 (3)	0.081 (3)	0.115 (4)	0.013 (3)	0.071 (3)	0.004 (3)
C18	0.082 (3)	0.117 (4)	0.074 (3)	0.019 (3)	0.047 (3)	0.038 (3)
C19	0.064 (3)	0.111 (4)	0.097 (4)	−0.026 (3)	0.047 (3)	−0.013 (3)
N21	0.0460 (15)	0.0488 (17)	0.0530 (16)	0.0030 (13)	0.0278 (14)	0.0065 (13)
C21	0.052 (2)	0.060 (2)	0.054 (2)	0.0076 (17)	0.0246 (18)	0.0103 (17)
C22	0.046 (2)	0.074 (3)	0.071 (3)	0.0149 (19)	0.0288 (19)	0.013 (2)
C23	0.048 (2)	0.075 (2)	0.058 (2)	−0.0019 (19)	0.0309 (19)	−0.0028 (19)
C24	0.0450 (18)	0.055 (2)	0.0395 (17)	−0.0068 (16)	0.0220 (15)	−0.0049 (15)
C25	0.0436 (18)	0.054 (2)	0.0477 (19)	0.0038 (16)	0.0230 (16)	0.0046 (16)
C26	0.063 (2)	0.071 (3)	0.049 (2)	−0.007 (2)	0.0303 (19)	0.0052 (19)
C27	0.118 (5)	0.159 (7)	0.151 (6)	0.059 (5)	0.084 (5)	0.103 (5)
C28	0.323 (12)	0.124 (6)	0.047 (3)	−0.086 (7)	0.063 (5)	−0.013 (3)
C29	0.094 (4)	0.110 (5)	0.124 (5)	−0.012 (4)	0.058 (4)	0.038 (4)

### Geometric parameters (Å, º)

**Table:**

Cd1—N2	2.305 (3)	C18—H18A	0.9600
Cd1—N1	2.322 (3)	C18—H18B	0.9600
Cd1—N21	2.339 (3)	C18—H18C	0.9600
Cd1—N11	2.368 (3)	C19—H19A	0.9600
Cd1—S2^i^	2.7412 (10)	C19—H19B	0.9600
Cd1—S1^ii^	2.7513 (10)	C19—H19C	0.9600
N1—C1	1.151 (4)	N21—C21	1.330 (5)
C1—S1	1.644 (4)	N21—C25	1.339 (4)
S1—Cd1^i^	2.7513 (10)	C21—C22	1.375 (5)
N2—C2	1.154 (4)	C21—H21	0.9300
C2—S2	1.644 (4)	C22—C23	1.374 (6)
S2—Cd1^ii^	2.7412 (10)	C22—H22	0.9300
N11—C11	1.314 (5)	C23—C24	1.385 (5)
N11—C15	1.319 (5)	C23—H23	0.9300
C11—C12	1.378 (5)	C24—C25	1.384 (4)
C11—H11	0.9300	C24—C26	1.518 (5)
C12—C13	1.373 (5)	C25—H25	0.9300
C12—H12	0.9300	C26—C28	1.492 (7)
C13—C14	1.379 (5)	C26—C27	1.514 (7)
C13—C16	1.527 (5)	C26—C29	1.518 (6)
C14—C15	1.379 (5)	C27—H27A	0.9600
C14—H14	0.9300	C27—H27B	0.9600
C15—H15	0.9300	C27—H27C	0.9600
C16—C19	1.519 (6)	C28—H28A	0.9600
C16—C18	1.525 (6)	C28—H28B	0.9600
C16—C17	1.537 (6)	C28—H28C	0.9600
C17—H17A	0.9600	C29—H29A	0.9600
C17—H17B	0.9600	C29—H29B	0.9600
C17—H17C	0.9600	C29—H29C	0.9600
			
N2—Cd1—N1	179.28 (11)	H18A—C18—H18B	109.5
N2—Cd1—N21	90.40 (11)	C16—C18—H18C	109.5
N1—Cd1—N21	89.39 (11)	H18A—C18—H18C	109.5
N2—Cd1—N11	93.03 (11)	H18B—C18—H18C	109.5
N1—Cd1—N11	87.23 (11)	C16—C19—H19A	109.5
N21—Cd1—N11	175.34 (11)	C16—C19—H19B	109.5
N2—Cd1—S2^i^	87.88 (8)	H19A—C19—H19B	109.5
N1—Cd1—S2^i^	91.43 (8)	C16—C19—H19C	109.5
N21—Cd1—S2^i^	90.86 (8)	H19A—C19—H19C	109.5
N11—Cd1—S2^i^	92.42 (8)	H19B—C19—H19C	109.5
N2—Cd1—S1^ii^	92.89 (8)	C21—N21—C25	117.4 (3)
N1—Cd1—S1^ii^	87.79 (8)	C21—N21—Cd1	119.6 (2)
N21—Cd1—S1^ii^	87.21 (8)	C25—N21—Cd1	123.0 (2)
N11—Cd1—S1^ii^	89.46 (8)	N21—C21—C22	122.5 (4)
S2^i^—Cd1—S1^ii^	177.92 (3)	N21—C21—H21	118.8
C1—N1—Cd1	163.7 (3)	C22—C21—H21	118.8
N1—C1—S1	178.0 (3)	C23—C22—C21	118.8 (4)
C1—S1—Cd1^i^	98.96 (11)	C23—C22—H22	120.6
C2—N2—Cd1	161.9 (3)	C21—C22—H22	120.6
N2—C2—S2	178.6 (3)	C22—C23—C24	120.7 (3)
C2—S2—Cd1^ii^	100.68 (11)	C22—C23—H23	119.6
C11—N11—C15	115.2 (3)	C24—C23—H23	119.6
C11—N11—Cd1	121.6 (2)	C25—C24—C23	115.6 (3)
C15—N11—Cd1	122.9 (2)	C25—C24—C26	122.4 (3)
N11—C11—C12	124.1 (4)	C23—C24—C26	122.0 (3)
N11—C11—H11	118.0	N21—C25—C24	124.9 (3)
C12—C11—H11	118.0	N21—C25—H25	117.5
C13—C12—C11	121.3 (4)	C24—C25—H25	117.5
C13—C12—H12	119.3	C28—C26—C27	110.3 (6)
C11—C12—H12	119.3	C28—C26—C24	108.3 (4)
C12—C13—C14	114.3 (3)	C27—C26—C24	111.8 (3)
C12—C13—C16	122.0 (3)	C28—C26—C29	109.6 (5)
C14—C13—C16	123.6 (3)	C27—C26—C29	105.6 (5)
C13—C14—C15	120.7 (4)	C24—C26—C29	111.2 (4)
C13—C14—H14	119.6	C26—C27—H27A	109.5
C15—C14—H14	119.6	C26—C27—H27B	109.5
N11—C15—C14	124.3 (4)	H27A—C27—H27B	109.5
N11—C15—H15	117.8	C26—C27—H27C	109.5
C14—C15—H15	117.8	H27A—C27—H27C	109.5
C19—C16—C18	109.7 (4)	H27B—C27—H27C	109.5
C19—C16—C13	111.2 (3)	C26—C28—H28A	109.5
C18—C16—C13	107.7 (3)	C26—C28—H28B	109.5
C19—C16—C17	108.5 (4)	H28A—C28—H28B	109.5
C18—C16—C17	108.5 (4)	C26—C28—H28C	109.5
C13—C16—C17	111.3 (3)	H28A—C28—H28C	109.5
C16—C17—H17A	109.5	H28B—C28—H28C	109.5
C16—C17—H17B	109.5	C26—C29—H29A	109.5
H17A—C17—H17B	109.5	C26—C29—H29B	109.5
C16—C17—H17C	109.5	H29A—C29—H29B	109.5
H17A—C17—H17C	109.5	C26—C29—H29C	109.5
H17B—C17—H17C	109.5	H29A—C29—H29C	109.5
C16—C18—H18A	109.5	H29B—C29—H29C	109.5
C16—C18—H18B	109.5		

Symmetry codes: (i) *x*, *y*+1, *z*; (ii) *x*, *y*−1, *z*.
