# Supplementary figures and images for: Crystal structure of catena-poly[[(3-tert-butyl­pyridine-κN)(4-tert-butyl­pyridine-κN)cadmium]-di-μ-thio­cyanato-κ2 N:S;κ2 S:N]
Source: Acta Crystallogr Sect E Struct Rep Online. 2014 Nov 19;70(Pt 12):m403–4. doi: 10.1107/S1600536814024647 (PMC4257453; doi:10.1107/S1600536814024647)

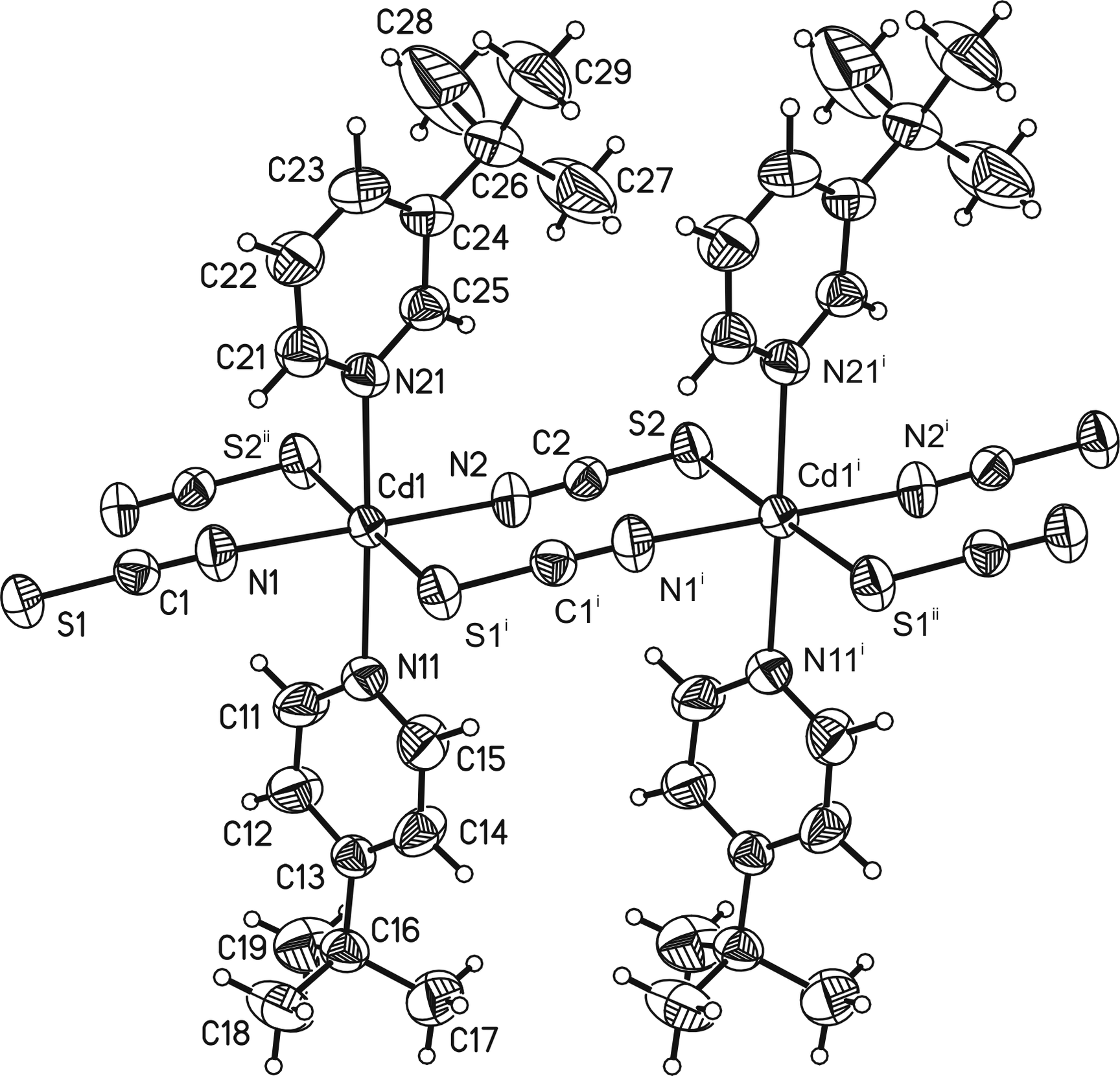

Supplement: Supplementary file 3 [file e-70-0m403-fig1.tif]
